# Phlebotomine sand fly–borne pathogens in the Mediterranean Basin: Human leishmaniasis and phlebovirus infections

**DOI:** 10.1371/journal.pntd.0005660

**Published:** 2017-08-10

**Authors:** Martina Moriconi, Gianluca Rugna, Mattia Calzolari, Romeo Bellini, Alessandro Albieri, Paola Angelini, Roberto Cagarelli, Maria P. Landini, Remi N. Charrel, Stefania Varani

**Affiliations:** 1 Department of Experimental, Diagnostic and Specialty Medicine, University of Bologna, Bologna, Italy; 2 Modena Unit, Istituto Zooprofilattico Sperimentale della Lombardia e dell'Emilia Romagna, Brescia, Italy; 3 Laboratory of Entomology, Istituto Zooprofilattico Sperimentale della Lombardia e dell'Emilia Romagna, Reggio Emilia, Italy; 4 Department of Medical and Veterinary Entomology, Centro Agricoltura Ambiente “G. Nicoli,” Crevalcore, Italy; 5 Public Health Authority, Emilia-Romagna Region, Bologna, Italy; 6 Istituto di Ricovero e Cura a carattere scientifico, Istituto Ortopedico Rizzoli, Bologna, Italy; 7 UMR “Emergence des Pathologies Virales” (EPV: Aix-Marseille Univ-IRD 190-Inserm 1207-EHESP), Marseille, France; 8 Fondation IHU Mediterranee Infection, APHM Public Hospitals of Marseille, Marseille, France; 9 Unit of Clinical Microbiology, Regional Reference Centre for Microbiological Emergencies (CRREM), St. Orsola-Malpighi University Hospital, Bologna, Italy; The Faculty of Medicine, The Hebrew University of Jerusalem, ISRAEL

## Abstract

Pathogens transmitted to humans by phlebotomine sand flies are neglected, as they cause infectious diseases that are not on the priority list of national and international public health systems. However, the infections caused by protozoa of the *Leishmania* genus and viruses belonging to the *Phlebovirus* genus (family Phenuiviridae)—the most significant group of viruses transmitted by sand flies—have a relevant role for human pathology. These infections are emerging in the Mediterranean region and will likely spread in forthcoming decades, posing a complex threat to human health. Four species and 2 hybrid strains of *Leishmania* are pathogenic for humans in the Mediterranean Basin, with an estimated annual incidence of 239,500–393,600 cases of cutaneous leishmaniasis and 1,200–2,000 cases of visceral leishmaniasis. Among the phleboviruses, Toscana virus can cause neuroinvasive infections, while other phleboviruses are responsible for a typical “3-day fever”; the actual incidence of *Phlebovirus* infections in the Mediterranean area is unknown, although at least 250 million people are exposed. Here, we reviewed the current literature on epidemiology of sand fly–borne infections in the Mediterranean Basin, with a focus on humans. Our analysis indicates the need for increased public health activities directed to determine the disease burden of these infections as well as to improve their surveillance. Among the emerging challenges concerning sand fly–borne pathogens, the relationships between sand fly–borne protozoa and viruses should be considered in future studies, including epidemiological links between *Leishmania* and phleboviruses as well as the conditional capacity for these pathogens to be involved in interactions that may evolve towards increased virulence.

## Introduction

Pathogens transmitted to humans and animals by phlebotomine sand flies are relatively neglected, as they cause infectious diseases that are not on the priority list of national and international public health agencies in Europe. However, these diseases are emerging in southern Europe [1]. Among sand fly–borne pathogens, protozoa of the *Leishmania* genus and viruses belonging to the *Phlebovirus* genus (order Bunyavirales, family Phenuiviridae)—the most significant group of viruses transmitted by sand flies—contribute significantly to human pathologic disease. Four recognized species and 2 hybrid strains of *Leishmania* are known to be pathogenic for humans in the Mediterranean region, with an estimated annual incidence of 239,500–393,600 cases of cutaneous leishmaniasis (CL) and 1,200–2,000 cases of visceral leishmaniasis (VL) [2]. Among the phleboviruses, Toscana virus (TOSV) can cause neuroinvasive infections, while other phleboviruses are responsible for a typical “3-day fever” or “pappataci fever;” the actual incidence of *Phlebovirus* infections in the Mediterranean area is unknown. However, at least 250 million people are potentially exposed [3]. The aim of this study is to provide an overview of the epidemiology of sand fly–borne infections in the Mediterranean Basin, with particular focus on humans.

## Methods

A comprehensive literature search was conducted on the distribution of *Leishmania* and *Phlebovirus* species responsible for human infection in the Mediterranean region. An additional search was dedicated to identifying the reservoir(s) of *Leishmania* and *Phlebovirus*. We analyzed all articles listed in the PubMed database in English, French, Italian, and Spanish between 1980 and December 2016, when the final search was conducted. The search terms used included leishmanial species, phlebovirus species, reservoir species, and countries included in the study. Titles relevant to the scope of this review were obtained in full text and selected for inclusion. Unpublished data were not considered. Data on the presence of *Leishmania* and/or *Phlebovirus* detection were reported on a geographic information system (GIS) at country level. Maps were created in the open source GIS software, QGIS 2.12 (www.qgis.org).

## Characteristics of phlebotomine sand fly–borne pathogens (*Leishmania* and *Phlebovirus*)

### Leishmania

The genus *Leishmania* is composed of protozoa belonging to the Trypanosamatidae family, order Kinetoplastida. Within the genus, there are 2 subgenera of medical importance that were created on the basis of the parasite developmental patterns within the sand fly gut: *Leishmania* (all development anterior to the pylorus) and *Viannia* (developing in the hindgut and pylorus) [4]. On the basis of multilocus enzyme electrophoresis (MLEE), from which the current *Leishmania* taxonomy originates, leishmanial species are grouped within each subgenus into so-called “species complexes” or “complexes,” which are distributed in countries with a subtropical climate [5].

These protozoa have a digenetic life cycle, requiring a susceptible vertebrate host and a permissive insect vector, which allow their transmission [4]. The blood-feeding females of phlebotomine sand flies are considered the only natural vectors of leishmanial species. The genus *Phlebotomus* includes species responsible for the transmission of *Leishmania* in the Old World, while *Lutzomyia* species are responsible for the transmission of the parasite in the New World [4].

The morphological forms of these protozoa are: (1) an extracellular flagellated promastigote that may be found in sand fly gut and (2) an obligate intracellular nonflagellated amastigote, typically residing in monocyte macrophages of the mammalian host [4].

About 20 *Leishmania* species are known to be pathogenic for humans [6] and are differently distributed in the New and Old Worlds [5]. The species circulating in the Mediterranean region include *L*. *donovani* and *L*. *infantum*, causing VL, and *L*. *tropica* and *L*. *major*, which, together with dermotropic *L*. *infantum* strains and *L*. *infantum/L*. *donovani* hybrid, cause CL [4, 7–9]. A classification of human pathogenic *Leishmania* species circulating in the Mediterranean region is presented in Table 1.

**Table 1:** Leishmanial species circulating in the Mediterranean region and pathogenic for humans.

Genus	Subgenus	Complex	Species	Geographical distribution
*Leishmania*				
	*Leishmania*			
		*L*. *major* complex		
			*L*. *major*	North Africa, the Middle East, and the Balkans
		*L*. *tropica* complex		
			*L*. *tropica*	North Africa, the Middle East, and Greece
			(*L*. *killicki*^a^)	North Africa
		*L*. *donovani* complex		
			*L*. *donovani*	Cyprus and Turkey
			*L*. *infantum*	Southern Europe, North Africa, and the Middle East
			(*L*. *archibaldi*^b^)	Lebanon, Tunisia, and Italy (rare isolates)

^a^ *L*. *tropica* subpopulation circulating in North Africa,

^b^ multilocus enzyme electrophoresis (MLEE)-defined species, not supported by phylogenetic studies based on sequence analysis. *L*. *archibaldi* has a restricted geographical range, essentially limited to East Africa [10]. Nevertheless, rare isolates were identified in the Mediterranean region, specifically in Lebanon [11], Tunisia, and Italy [12].

Survival of the *Leishmania* parasites depends on successful zoonotic or anthroponotic transmission and on suitable environment and climate. Although the areas in which different leishmanial species are diffused appear well defined, the risk for introduction of exotic species through travel or migration should be considered. The probability that new species could enter into a local transmission cycle depends on various factors, including the competence of sand flies for different leishmanial species; the presence of a suitable reservoir; and the contact between sand flies, reservoirs, and humans [1]. Considering these aspects, the introduction of zoonotic *L*. *major* to Europe is regarded as unlikely, as the fat sand rats and the gerbils—the natural animal reservoirs of *L*. *major*—live in the desert or semidesert areas of the African and Asian continents not found on the European continent [13]. On the contrary, the risk of introduction of anthroponotic *L*. *tropica* and *L*. *donovani* to Europe is not negligible because of the diffusion in various European countries of the respective vectors, *P*. *sergenti* and *P*. *tobbi* [14, 15].

CL cases caused by *L*. *tropica* were recorded in Greece for the first time in 1984 [16]. However, Garifallou et al. reported on the first biochemical typing of CL agents, while CL cases had been documented in Greece long before the 80s [17]. Strain typing indicated that the Greek strain of *L*. *tropica* closely resembled *L*. *tropica* strains from the Middle East and Asia [16].

The report of autochthonous cases of CL and VL caused by *L*. *donovani* (MON-37 zymodeme) in Cyprus [18] confirms the dynamic nature of this parasitic infection. MLEE analysis showed that the Cypriotic *L*. *donovani* MON-37 is closely related to 2 different strains belonging to *L*. *donovani* MON-309 zymodeme (Çukurova strains) and to *L*. *donovani* MON-308 zymodeme, both identified in Turkey and transmitted by *P*. *tobbi* [8]. Strains of *L*. *donovani* belonging to the MON-37 zymodeme are geographically the most widespread and give the impression of constituting a homogeneous group. However, multilocus microsatellite typing (MLMT) analysis revealed that MON-37 strains from Cyprus are clearly different compared with MON-37 strains from the Indian subcontinent, the Middle East, China, and East Africa [19]. A distinct MLMT study placed the Turkish strains and the Cypriotic strain in a subclade of a newly discovered group (Turkey/Cyprus non-MON-1 zymodeme group) within the *L*. *donovani* complex [8], suggesting the diffusion of these strains over neighboring areas in the European continent.

The reproduction mode (clonality versus sexuality) of the *Leishmania* parasite is still under debate, but the existence of natural hybrid strains suggests that the exchange of genetic material does occur. Recently, whole genome sequencing demonstrated that the çukurova strain is the progeny of a single outcrossing event between 1 parent related to *L*. *infantum* and an as-yet-unidentified parent belonging to the *L*. *donovani* complex [9]. Moreover, 2 hybrid strains produced by *L*. *infantum* and *L*. *major* were isolated in Portugal from immunocompromised patients suffering from VL, indicating that close phylogenetic association is not a necessary prerequisite for genetic exchange [20]. In addition, molecular techniques show that these chimeric strains contain the complete genome of both *L*. *infantum* and *L*. *major*. Genetic exchange may have potential implications such as emergence of virulent strains or increasing fitness [21, 22]. Indeed, *L*. *infantum*/*L*. *major* hybrid is capable of replicating in *P*. *papatasi* [22], while *L*. *infantum*/*L*. *donovani* hybrid replicates in *P*. *tobbi* and *P*. *perniciosus* [15], suggesting that hybrid strains may circulate in infected sand flies.

### Phlebovirus

The genus *Phlebovirus* belongs to the family Phenuiviridae, order Bunyavirales [3]. Phleboviruses are enveloped, spherical viruses. The genome is a negative-sense, single-stranded RNA. The 3 RNA segments designated as large (L), medium (M), and small (S) encode the RNA-dependent RNA polymerase, the envelope glycoproteins, and the nucleoprotein, respectively. The single-stranded RNA segments have high mutation rates due to the lack of proofreading activity of the viral polymerase, which may result in genetic drift following point mutation, while the 3 genome segments are involved in reassortment/recombination, leading to the generation of new viruses [3].

The genus *Phlebovirus* includes 9 species; 70 antigenically distinct viruses are segregated into the 9 species, while 33 viruses are not yet attributed to one of these species [23].

**Fig 1:**
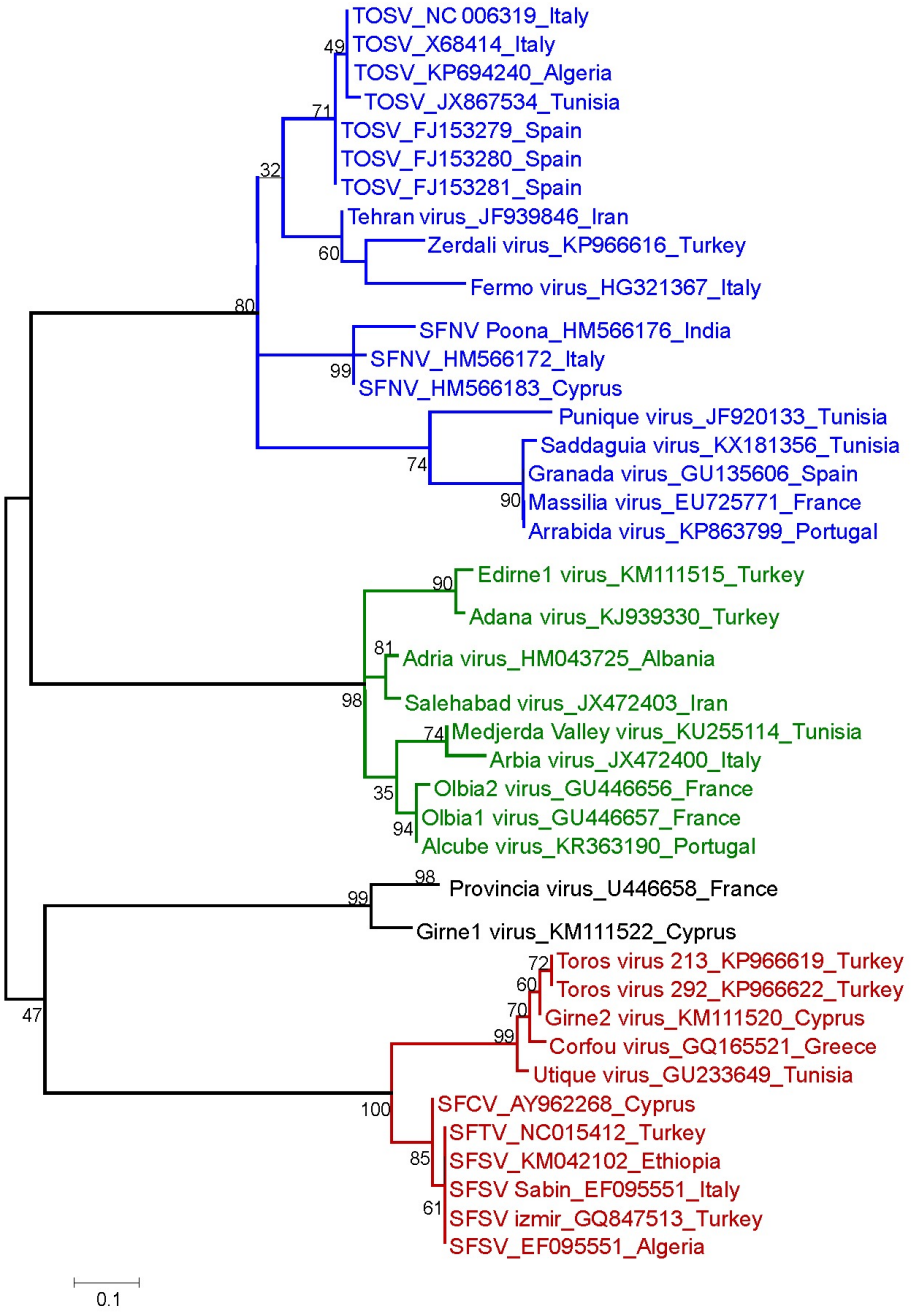
Phleboviruses phylogeny reconstruction. Phylogenetic relationships between selected Old World sand fly–borne phleboviruses based on partial large (L) RNA sequences; phylogenetic relationships of selected amino acid sequences were inferred by using the maximum likelihood method based on the Jones-Taylor-Thornton (JTT) model gamma distributed with invariant sites [35]. A discrete gamma distribution was used to model evolutionary-rate differences among sites [5 categories]. The analysis involved 44 sequences. All positions with less than 95% site coverage were eliminated so that there was a total of 55 positions in the final dataset. Evolutionary analysis was conducted in MEGA6 using 500 bootstrap pseudoreplications [35].

In the Mediterranean area, phleboviruses transmitted by sand flies belong to 1 of the following groups: (i) 2 International Committee on Taxonomy of Viruses (ICTV)-recognized species, Sandfly fever Naples (SFNV) and Salehabad, (ii) 2 tentative species (Sandfly fever Sicilian virus [SFSV] and Corfou virus) [23]. Furthermore, Karimabad virus was erroneously listed in the Sandfly fever Naples species as demonstrated by Palacios et al. [24] and should be listed as a tentative species. A large number of new sand fly–borne phleboviruses were recently described, all of them belong to species/groups listed as (i) or (ii) (Table 2). A phlebovirus phylogeny reconstruction is depicted in Fig 1.

**Table 2:** Pathogenic (or potentially pathogenic) phleboviruses for humans in the Mediterranean region.

Family	Genus	Species or tentative species (ts)	Virus	Reference
**Bunyaviridae**	*Phlebovirus*	Sandfly fever Naples	Sandfly fever Naples virus (SFNV) Toscana virus (TOSV) Massilia virus (MASV) Tehran virus (TEHV) Granada virus (GRV) Punique virus (PUNV) Fermo virus Saddaguia virus (SADV) [md/ni] Arrabida virus Zerdali virus (ZERV)	[24] [24] [24] [24] [24] [24] [25] [26] [27] [28]
		Salehabad	Salehabad virus (SALV) Arbia virus (ARBV) Adria virus (ADRV) [md/ni] Alcube virus Edirne virus [md/ni] Adana virus (ADAV) Medjerda Valley virus (MVV)	[29] [29] [29] [30] [31] [32] [33]
		Sandfly fever Sicilian (ts)	Sandfly fever Sicilian virus (SFSV) Cyprus virus (SFCV) Turkey virus (SFTV) Utique virus [md/ni]	[3] [3] [3] [3]
		Corfou (ts)	Corfou virus (CFUV) Toros virus (TORV) Sicilian-like virus Girne 1 virus [md/ni] Girne 2 virus [md/ni] Olbia virus [md/ni] Provencia virus [md/ni]	[3] [28] [3] [31] [31] [34] [34]
		Karimabad (ts)	Karimabad virus (KARV)	[24]

[md/ni], molecular detection only, no virus isolation

SFSV and SFNV were first isolated from the sera of sick soldiers in Egypt in 1943 and Naples in 1944, respectively [3]. TOSV was first isolated in the Tuscany region of central Italy from *P*. *perniciosus* and *P*. *perfiliewi* in 1971 and 12 years later was detected in the central nervous system (CNS) of patients with meningitis [3]. Because of the geographic spread of TOSV in southern Europe and surrounding countries and the growing number of human cases [36], TOSV is recognized as an emerging pathogen and one of the most common causes of summer meningitis in the Mediterranean Basin.

On the basis of sequence analysis of the M RNA segment, different clusters of TOSV were clearly distinguished and named TOSV lineages A, B, and C (S1 Table).

## The reservoir hosts

### Reservoirs of *Leishmania*

The ecological system in which *Leishmania* is maintained is composed of vector and vertebrate reservoirs [4]. Usually, there is 1 principal reservoir host, which differs on the basis of the *Leishmania* infecting species and the infection focus (Table 3). Other susceptible mammals may become infected as minor or incidental hosts. Minor hosts may play a role in parasite maintenance, while incidental hosts are infected mammals without this role.

**Table 3:** Transmission cycle, geographical distribution, and proven/suspected reservoirs of *Leishmania* in the Mediterranean region.

Transmission cycle	Species	Distribution	Reservoirs^a^^,^^b^	References
**Zoonotic**	*Leishmania* *infantum*	Mediterranean Basin	Dog^a^ (*Canis lupus familiaris)*, cat^b^ (*Felis catus*), red fox^b^ (*Vulpes vulpes*), golden jackal^b^ (*Canis aureus*), wolf ^b^ (*Canis lupus*), badger^b^ (*Meles meles*), common genet^b^ (*Genetta genetta*), pine marten^b^ (*Martes martes*), wild cat^b^ (*Felis silvestris*), Iberian lynx^b^ (*Lynx pardinus*), Iberian hare^b^ (*Lepus granatensis*), wild rabbit^b^ (*Oryctolagus cuniculus*), black rat^b^ (*Rattus rattus*), Norwegian rat^b^ (*Rattus norvegicus*)	[6, 41–45]
	*L*. *major*	North Africa and the Middle East	Fat sand rat^a^ (*Psammomys obesus*), gerbils^a^ (*Meriones* spp.), Algerian hedgehog^b^ (*Ateleris algirus*), desert hedgehog^b^ (*Paraechinus aethiopicus*), cat^b^ (*Felis catus*)	[13, 46–48]
	*L*. *tropica*	North Africa and the Middle East	Rock hyrax^b^ (*Procavia capensis*), North African gundi^b^ (*Ctenodactylus gundi*), cat^b^ (*Felis catus*)	[38–40, 48, 49]
**Anthroponotic**	*L*. *donovani*	Cyprus and Turkey	Human^c^ (*Homo sapiens*)	[18, 50]
	*L*. *tropica*	North Africa and the Middle East	Human (*Homo sapiens*)	[4]

^a^ proven reservoir: direct or indirect evidence of transmission to the target population (humans),

^b^ suspected reservoir: evidence of *Leishmania* spp. infection (culture, serological, or molecular methods) but no evidence of transmission to the target population (humans),

^**c**^ anthroponotic cycle has not yet been confirmed.

#### Humans

Humans are directly involved as principal reservoir hosts in VL caused by *L*. *donovani* and CL caused by *L*. *tropica* [4]. While the role of patients with clinical symptoms as reservoirs is clear, the role of asymptomatically infected individuals in the transmission of infection is currently unknown. Asymptomatic carriers exhibit low parasitemia, thus being less infective than symptomatic individuals [4]. Nevertheless, the huge numbers of asymptomatically infected individuals constitute a possible source of infection [37].

#### Animals

Wild or domestic animals are the main reservoirs of *L*. *infantum* and *L*. *major*. Recently, some animal species have been implicated as reservoirs for *L*. *tropica* [4, 6, 38–40] (Table 3).

Domestic dogs (*Canis lupus familiaris*) are the primary domestic reservoir hosts of *L*. *infantum*. After infection, dogs may show clinical manifestations with widespread dissemination of parasites both in viscera and dermis, making this animal an excellent source of infection for vectors; infection efficiency for sand flies increases with the increasing clinical severity [51]. Nevertheless, asymptomatic seropositive dogs may play a role in infection maintenance in the domestic transmission cycle [51].

The epidemiological role of other domestic animals, such as cats (*Felis catus*), is still controversial [42], while evidence of infection with *L*. *infantum* in Mediterranean wildlife has been reported in a range of carnivores, rodents, and lagomorphs [52].

Between 2009 and 2012, an outbreak of human leishmaniasis caused by *L*. *infantum* occurred in Madrid with 446 cases [53]; hares were identified as the reservoir of *Leishmania* during the epidemic. In fact, no increase in *Leishmania* seroprevalence was observed in dogs during the human outbreak, while a high number of asymptomatic Iberian hares (*Lepus granatensis*) infected with *L*. *infantum* were found [44]. The demonstration that naturally infected hares can transmit parasites to sand flies combined with molecular and ecoepidemiological data infers that Iberian hares can be considered as wild reservoirs of *Leishmania* in Spain [44].

*L*. *major* mainly causes a zoonotic infection, with infected wild rodents regarded as reservoirs [54] (Table 3). In the Mediterranean Basin, the main reservoir species is the fat sand rat (*Psammomys obesus*), a rodent that is very abundant in arid pre-Saharan areas, from Mauritania to the Middle East. In addition, gerbils (*Meriones* spp.) play a role in the maintenance of the circulation of *L*. *major*. Among the others, *Meriones shawi* is the reservoir host of *L*. *major* in some parts of Tunisia, Morocco, and Algeria [13], while *M*. *tristrami* and *M*. *guentheri* are suspected as the main reservoir hosts of *L*. *major* in a focus of human CL in Israel [46]. The epidemiology and control strategies of human leishmaniasis largely depend on the distribution and abundance of these rodents, which in turn are influenced by agricultural development and ecological changes. Migratory habits of some rodent species (i.e., *Meriones* spp.) could also contribute to the spread of *L*. *major* from endemic foci towards neighboring areas [13]. In addition, other mammals can be involved in the maintenance of the transmission cycle of *L*. *major*; natural infection in 2 species of hedgehogs (*Ateleris algirus* and *Paraechinus aethiopicus*) was recently reported from Algeria, suggesting their involvement in the parasite cycle [47].

### Reservoirs of phleboviruses

No reservoir host has been defined with certainty for phleboviruses. Concerning TOSV infection, it is unlikely that humans play this role because of the transient viremia occurring in infected patients [36]; competent sand fly species might act as reservoirs in the viral cycle [6, 55, 56]. In support of this hypothesis, male sand flies were found to be infected by TOSV in nature, while transovarial transmission was experimentally demonstrated as well as venereal transmission from infected *P*. *pernicious* males to uninfected females. However, the progressive decrease in viral infection rates observed from generation to generation in sand fly colonies suggests that TOSV cannot be maintained indefinitely by vertical or venereal transmission [6]. Consequently, the existence of additional reservoirs should be considered.

Only a few studies were conducted on animals to verify their role as reservoirs of phleboviruses. In 1984, TOSV was isolated from a bat in Italy, while the high frequency of viral RNA and specific antibodies for TOSV in the canine population in Tunisia and Turkey suggests that dogs can act as TOSV reservoirs [57, 58]. However, this merits further exploration in other geographic areas and through experimental studies. TOSV antibodies were also revealed in horses, cats, dogs, sheep, pigs, cows, and goats in Spain [59] and in dogs living in Corsica [60] and Algeria [61]. Although there is no direct evidence for infection of vertebrates by sand fly–borne phleboviruses other than TOSV, high rates of neutralizing antibodies in various mammals demonstrate high exposure to phleboviruses in Tunisia and Turkey [32, 58].

## Human infection caused by phlebotomine sand fly–borne pathogens in the Mediterranean Basin

Clinical syndromes caused by sand fly–borne pathogens are only the tip of the iceberg; asymptomatic infected carriers are frequent and likely more abundant in rural areas, where vectors are numerous [37]. Human seroprevalence for *Leishmania* and *Phlebovirus* in the countries of the Mediterranean Basin is reported in S2 Table.

The clinical spectrum of human leishmaniasis ranges from asymptomatic infection to 3 main clinical syndromes: VL, CL, and mucosal leishmaniasis (ML) [4]. Clinical manifestations are the result of the relationship between the leishmanial species and the host and are partially dependent on strain differences, making some species more adapted to target the skin and others to invade visceral organs [62]. VL is the most severe form of the disease, and it is fatal if untreated; the target organs of VL are the spleen, the liver, the bone marrow, and the lymph nodes, where parasites proliferate in the cells of the mononuclear phagocytic system [4, 63]. CL is characterized by single or multiple ulcerative or nodular lesions in exposed areas of the body; *L*. *tropica* mainly causes a single lesion, while *L*. *major* is often characterized by multiple lesions [5, 64]. ML pathophysiology in the Mediterranean region remains unclear as it is usually not associated with previous CL [65]. The most frequent localizations are larynx, pharynx, mouth, and nose, and the lesions are polypoid, infiltrative, ulcerative, or nodular [65–69].

SFSV and SFNV cause a typical “3-day fever” or “pappataci fever,” while TOSV displays a strong neurotropism responsible for acute meningitis and meningoencephalitis [36]. Nevertheless, most cases of TOSV infection present as asymptomatic cases or as a flu-like syndrome [70]. In addition, an outbreak of febrile syndrome was observed in Greek soldiers stationed in Cyprus in 2002; molecular investigation confirmed the presence of a new phlebovirus provisionally named Sandfly fever Cyprus virus [71]. Finally, Adria virus was detected in the blood of a Greek child with febrile seizure [72] and Sandfly fever Turkey virus was isolated from the cerebrospinal fluid of a Turkish patient with encephalitis [73].

**Fig 2:**
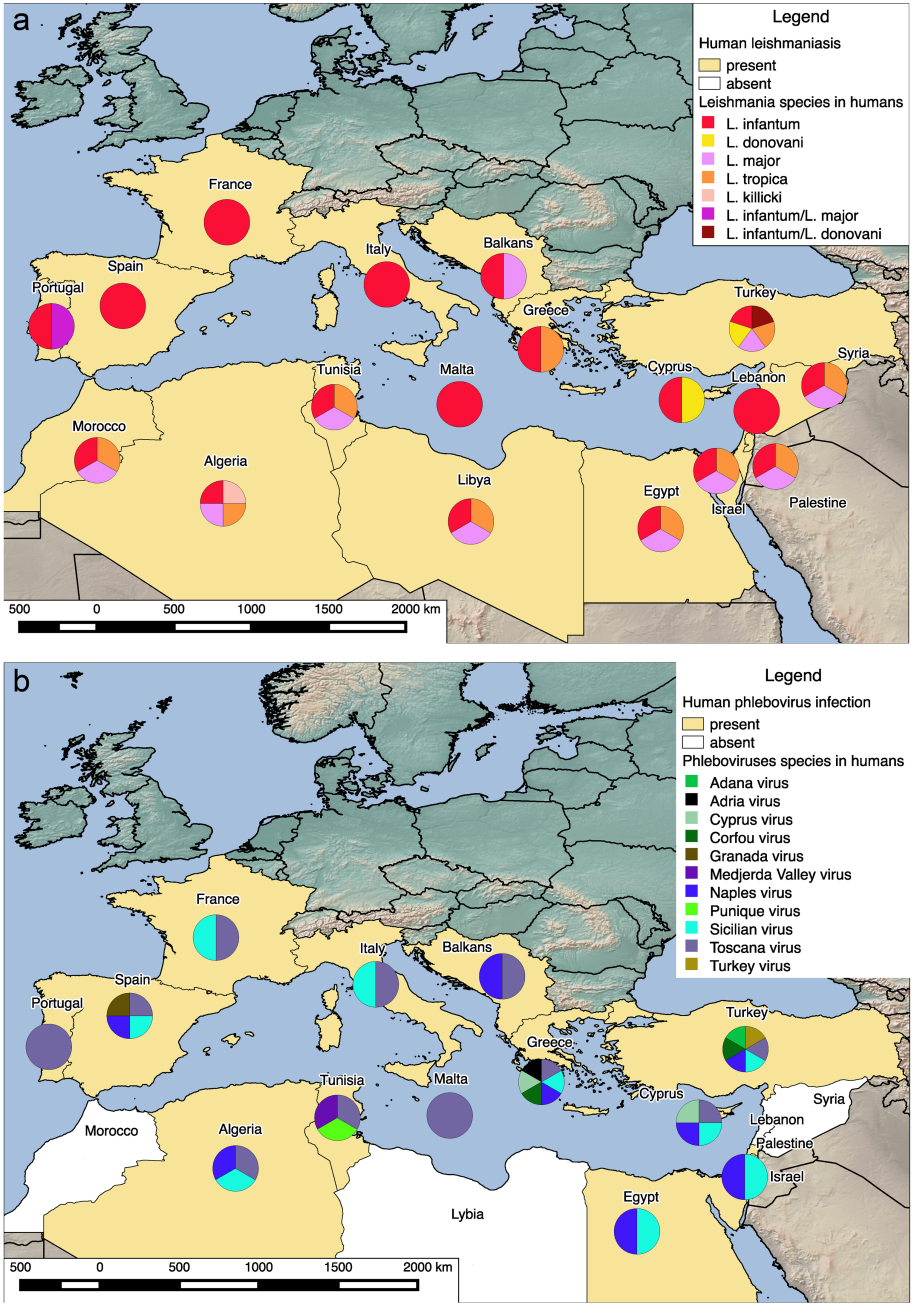
Distribution of human infections caused by sand fly–transmitted pathogens in the Mediterranean region using spatial methods. **(a)** Autochthonous human leishmaniasis in the Mediterranean area. Countries where human leishmaniasis was diagnosed by serological tests and/or *Leishmania* isolation and/or PCR method are depicted in yellow. Diagrams show *Leishmania* species identified and/or isolated in human cases. **(b)** Autochthonous human phlebovirus infection in the Mediterranean area. Countries where human phlebovirus infection was diagnosed by serological tests and/or *Phlebovirus* isolation and/or PCR method are depicted in yellow, while white areas represent countries where human phlebovirus infection was never observed. Diagrams show *Phlebovirus* species identified and/or isolated in human cases; where no diagram is present, identification of phleboviruses species was not performed. Maps were created using the open source software, QGIS 2.12.

Fig 2 and Table 4 report the distribution of human infections caused by *Leishmania* and phleboviruses in the Mediterranean region.

**Table 4:** Distribution of *Leishmania* and *Phlebovirus* responsible for human infection in Mediterranean region countries.

Country	Leishmaniasis			Phleboviral infection	
		Notifiable disease (Y/N)	References		References
Portugal	*Leishmania infantum* (CL and VL) *L*. *infantum/L*. *major* (VL)	Y	[20, 74]	TOSV	[75]
Spain	*L*. *infantum* (CL, VL and ML)	Y	[76]	TOSV	[77]
France^a^	*L*. *infantum* (CL, VL and ML)	Y	[78]	TOSV	[79]
Italy	*L*. *infantum* (CL and VL)	Y	[80]	TOSV	[81]
Greece	*L*. *infantum* (CL and VL) *L*. *tropica* (CL)	Y	[16, 82]	TOSV ADRV^b^	[46, 83]
The Balkans	*L*. *infantum* (CL and VL): Bosnia-Herzegovina, Croatia, Albania *L*. *major* (CL): Albania *L*. *infantum* (VL): Montenegro Serbia^c^	Y (Albania, Bosnia-Herzegovina, Croatia, Montenegro) nf^d^ (Serbia)	[2]	TOSV	[84, 85]
Cyprus	*L*. *infantum*^e^(CL and VL) *L*. *donovani*^e^ (CL and VL)	Y	[18, 86]	SFSV SFNV SFCV^f^	[71, 87, 88]
Malta	*L*. *infantum* (CL and VL)	Y	[89]	TOSV	[90]
Turkey	*L*. *tropica* (CL) *L*. *infantum* (CL^g^ and VL) *L*. *infantum/L*. *donovani* (CL^g^) *L*. *major* (CL) *L*. *donovani* (VL)	Y	[9, 91]	SFSV CFUV^h^ TOSV SFTV^i^ SFNV	[73, 92]
Syria	*L*. *infantum* (VL) ^j^^,^ ^k^ *L*. *tropica* (CL) *L*. *major* (CL)	N	[93, 94]	Not reported	
Lebanon	*L*. *infantum* (CL)	Y	[95]	Not reported	
Israel	*L*. *major* (CL) *L*. *tropica* (CL) *L*. *infantum* (CL^l^ and VL)	Y	[96–98]	Not reported	
Palestine	*L*.*major* (CL) *L*. *tropica* (CL) *L*. *infantum* (CL^l^ and VL)	Y	[64, 99–101]	Not reported	
Morocco	*L*. *infantum* (CL and VL) *L*. *tropica* (CL) *L*. *major* (CL)	Y	[102, 103]	Not reported	
Algeria	*L*. *major* (CL) *L*. *infantum* (CL and VL) *L*. *tropica* (CL) *L*. *killicki* (CL)	Y	[2, 103]	Not reported	
Tunisia	*L*. *infantum* (CL, VL and ML^m^) *L*. *major* (CL and ML) *L*. *tropica* (CL)	Y	[103–105]	TOSV	[106]
Libya	*L*. *infantum* (CL and VL) *L*. *major* (CL) *L*. *tropica* (CL)	Y	[103, 107]	Not reported	
Egypt	*L*. *infantum* (VL) *L*. *major* (CL) *L*. *tropica* (CL)	N	[2, 103]	SFNV	[108]

^a^The endemic area of leishmaniasis and phleboviral infection is restricted to southern France [1],

^b^ADRV, Adria virus,

^c^No identification of species was performed in Serbia,

^d^nf, not found,

^e^Two different transmission cycles are present in Cyprus: (1) a zoonotic cycle involving *L*. *infantum* MON-1 zymodeme, (2) a probable anthroponotic cycle involving *L*. *donovani* MON-37 zymodeme [86],

^f^SFCV, Sandfly fever Cyprus virus,

^g^ *L*. *infantum/L*. *donovani* hybrid causes CL in Adana/Turkey, while *L*. *infantum* causes CL in Hatay/Turkey [7],

^h^CFUV, Sandfly fever Corfou virus,

^i^SFTV, Sandfly fever Turkey virus,

^j^Circulation of *L*. *donovani* in Syria has been suggested but still not demonstrated [50],

^k^Syria exhibits the highest prevalence of CL in the Mediterranean region: 53,000 CL cases were reported in 2012 and 41,000 CL cases in the first 6 months of 2013 [94],

^l^ *L*. *donovani* is a suspected agent of CL in Israel and Palestine [2, 100],

^m^An emerging clinical form, ML of the lip, was recently observed in Tunisia [109]

**Abbreviations**: CL, cutaneous leishmaniasis; ML, mucosal leishmaniasis; N, no; SFNV, Sandfly fever Naples virus; SFSV, Sandfly fever Sicilian virus; TOSV, Toscana virus; VL, visceral leishmaniasis; Y, yes

## Effect of global changes on phlebotomine sand fly–borne pathogens

Environmental aspects such as climate change, increasing urbanization, economic development, and population movements for economic, social, or political reasons are causing changes in the epidemiology of vector-borne infections, as they lead to alteration in the range of vectors, reservoirs, and pathogens [4].

Man-made ecological changes including urbanization, spread of agriculture into semiarid lands, and the introduction of artificial irrigation systems can affect the incidence of sand flies [4, 110, 111]. Other human activities, such as tourism and pilgrimages from or to endemic areas, may contribute to the spread of exotic pathogens to areas that are already endemic for other sand fly–borne pathogens.

In disadvantaged areas, other factors contribute to the diffusion of sand fly–borne diseases. For example, poor domestic and peridomestic sanitary conditions may attract anthropophilic sand flies and may increase breeding sites of the vectors, while malnutrition and high incidence of HIV infection may result in inadequate immune response and higher clinical expression of infections.

In conflict areas, lack of or destruction of healthcare infrastructures may occur as well as massive population displacement, the latter potentially causing the introduction of nonimmune people into areas with endemic/enzootic disease or the emergence of pathogens in areas where infected individuals are displaced and where reservoirs and vectors coexist. As the most recent example, the Syrian refugee crisis has precipitated a catastrophic outbreak of CL, leading to hundreds of thousands of CL-affected people living in refugee camps in Lebanon or trapped in conflict zones [112]. The exodus of migrants from Syria has led to a dramatic increase of CL cases in southern Turkey and to the introduction of new leishmanial species, such as *L*. *major* [50]. Similar situations may also be unfolding in other conflict areas, such as Libya and Yemen [112].

Although different factors may influence the diffusion of sand fly–borne diseases, the range and density of vectors are considered the most important risk factors for the emergence of leishmaniasis and phlebovirus diseases in Mediterranean and continental Europe [14, 113]. The recent geographical expansion of phlebotomine vectors in the Mediterranean region has been attributed to ongoing climate changes and investigation on seasonal dynamics of vectors for *L*. *infantum* in Mediterranean Europe confirmed that temperature is a major determinant for the seasonal interval of sand fly activity [114]. Thus, global warming can affect the diffusion of sand flies and sand fly–borne diseases, as shown by the detection of sand flies in continental Europe, including Germany, Austria, Switzerland, and Slovakia [113, 115–117] and by the northward spread of leishmaniasis in Italy, respectively [6]. In addition, large areas of continental Europe that are unsuitable for sand fly species today are projected to change towards a climate that will support the survival and replication of these vectors [113].

Factors other than vector density can influence the presence and diffusion of sand fly–borne pathogens, including the replication rate of the pathogen, the vector biting rate, and host and reservoir availability [113]. Climate changes, indeed, can also influence the development cycle of *Leishmania* promastigotes in sand flies; increases in minimum temperature accelerate the parasitic life cycle [113, 118].

## Conclusions

Sand fly–borne pathogens such as *Leishmania* spp. and phleboviruses are currently emerging in southern Europe, posing a complex threat to human health. A number of factors can influence the epidemiology of sand fly–transmitted infections, including environmental and climate changes, poverty, mass migration, and conflicts. Concerning the spreading of *Leishmania*, changes in infected animal reservoirs are also important; the northward spread of human leishmaniasis in Italy following the detection of infected dogs in the northern part of the country as well as the recent outbreak in Spain with hares as reservoirs show that hosts play a major role in transmission cycle under changing conditions.

Our review highlights the fact that, despite their emergence, several aspects of the epidemiology of phlebotomine sand fly–borne infections are poorly studied. Among others, there is an urgent need for increased public health activities to ameliorate the surveillance of sand fly–borne infections, considering that underreporting of VL and CL is observed in countries with a compulsory notification system for leishmaniasis [119] and *Phlebovirus* infection are not subjected to notification. Diagnostic tools should also be implemented, focusing on molecular tests for rapid pathogen detection, while studies on the potential role of domestic and wild mammals as reservoir hosts should be carried out as well as continuous surveillance of canine leishmaniasis. There is also a need to continuously monitor vector distribution in endemic as well as nonendemic countries—activities that are so far scarce and scattered—with in-depth investigation into the vectorial capacity of the sand fly species identified in different areas. Among the emerging challenges concerning sand fly–borne pathogens, the relationships between sand fly–borne protozoa and viruses should be considered in future studies, including epidemiological links between *Leishmania* and phleboviruses as well as the conditional capacity for these pathogens to be involved in interactions that may evolve towards increased virulence.

**Key learning points**

- Pathogens transmitted to humans by phlebotomine sand flies such as *Leishmania* spp. and phleboviruses are currently emerging in southern Europe.
- Evidence of *Leishmania* hybrid strains suggests that the exchange of genetic material can occur in these parasites.
- Genomic data on newly detected sand fly–borne phleboviruses are available, which allows the development of novel molecular tests to evaluate the role of these viruses in human pathology.
- A number of environmental factors such as climate change, increasing urbanization, economic development, and population movements are causing changes in the epidemiology of sand fly**–**borne infections as the result of altering the accessible range of vectors, reservoirs, and pathogens.
- Massive population displacement from conflict areas can cause the introduction of nonimmune people into areas with endemic/enzootic disease or the emergence of pathogens in areas where infected individuals are displaced and where reservoirs and vectors coexist.

**Top 5 papers**

1. Alvar J, Vélez ID, Bern C, Herrero M, Desjeux P, Cano J, et al. Leishmaniasis worldwide and global estimates of its incidence. PLoS ONE. 2012;7(5):e35671.
2. Alkan C, Bichaud L, de Lamballerie X, Alten B, Gould EA, Charrel RN. Sandfly-borne phleboviruses of Eurasia and Africa: epidemiology, genetic diversity, geographic range, control measures. Antiviral Res. 2013;100(1):54–74.
3. Dujardin JC, Campino L, Cañavate C, Dedet JP, Gradoni L, Soteriadou K, et al. Spread of vector-borne diseases and neglect of Leishmaniasis, Europe. Emerg Infect Dis. 2008;14(7):1013–8.
4. Gradoni L. Epidemiological surveillance of leishmaniasis in the European Union: operational and research challenges. Euro Surveill. 2013;18(30):20539.
5. Depaquit J, Grandadam M, Fouque F, Andry PE, Peyrefitte C. Arthropod-borne viruses transmitted by Phlebotomine sand flies in Europe: a review. Euro Surveill. 2010 Mar;15(10):19507. PubMed PMID: 20403307. eng.

## Supporting information

S1 Table TOSV lineage distribution in the Mediterranean region. (DOCX)

S2 Table Human seroprevalence of *Leishmania* and phleboviruses in the Mediterranean region. (DOCX)
