# Supplementary material for: Phlebotomine sand fly–borne pathogens in the Mediterranean Basin: Human leishmaniasis and phlebovirus infections
Source: PLoS Negl Trop Dis. 2017 Aug 10;11(8):e0005660. doi: 10.1371/journal.pntd.0005660 (PMC5552025; doi:10.1371/journal.pntd.0005660)
Supplement: S1 Table — (DOCX) [file pntd.0005660.s001.docx]

**S1 Table.**  **TOSV lineage distribution in the Mediterranean Region.**

| TOSV lineage | Geographical distribution | Reference |
| --- | --- | --- |
| A | Italy  Tunisia  Algeria  France  Turkey | [1]  [2]  [3]  [4]  [5] |
| B | Spain  Portugal  Morocco  France  Turkey | [6]  [7]  [7]  [4]  [5] |
| C | Croatia  Greece | [8]  [9] |

**References**

1. Verani P, Ciufolini MG, Nicoletti L, Balducci M, Sabatinelli G, Coluzzi M, et al. [Ecological and epidemiological studies of Toscana virus, an arbovirus isolated from Phlebotomus]. Ann Ist Super Sanita. 1982;18(3):397-9.

2. Bichaud L, Dachraoui K, Piorkowski G, Chelbi I, Moureau G, Cherni S, et al. Toscana virus isolated from sandflies, Tunisia. Emerg Infect Dis. 2013;19(2):322-4.

3. Alkan C, Allal-Ikhlef AB, Alwassouf S, Baklouti A, Piorkowski G, de Lamballerie X, et al. Virus isolation, genetic characterization and seroprevalence of Toscana virus in Algeria. Clin Microbiol Infect. 2015;21(11):1040.e1-9.

4. Charrel RN, Izri A, Temmam S, Delaunay P, Toga I, Dumon H, et al. Cocirculation of 2 genotypes of Toscana virus, southeastern France. Emerg Infect Dis. 2007;13(3):465-8.

5. Dincer E, Gargari S, Ozkul A, Ergunay K. Potential animal reservoirs of Toscana virus and coinfections with Leishmania infantum in Turkey. Am J Trop Med Hyg. 2015;92(4):690-7.

6. Sanbonmatsu-Gámez S, Pérez-Ruiz M, Collao X, Sánchez-Seco MP, Morillas-Márquez F, de la Rosa-Fraile M, et al. Toscana virus in Spain. Emerg Infect Dis. 2005;11(11):1701-7.

7. Es-Sette N, Nourlil J, Hamdi S, Mellouki F, Lemrani M. First detection of Toscana virus RNA from sand flies in the genus *Phlebotomus* (Diptera: Phlebotomidae) naturally infected in Morocco. J Med Entomol. 2012;p. 1507-9.

8. Punda-Polić V, Mohar B, Duh D, Bradarić N, Korva M, Fajs L, et al. Evidence of an autochthonous Toscana virus strain in Croatia. J Clin Virol. 2012;55(1):4-7.

9. Papa A, Paraforou T, Papakonstantinou I, Pagdatoglou K, Kontana A, Koukoubani T. Severe encephalitis caused by Toscana virus, Greece. Emerg Infect Dis. 2014;20(8):1417-9.
