# Supplementary material for: Phlebotomine sand fly–borne pathogens in the Mediterranean Basin: Human leishmaniasis and phlebovirus infections
Source: PLoS Negl Trop Dis. 2017 Aug 10;11(8):e0005660. doi: 10.1371/journal.pntd.0005660 (PMC5552025; doi:10.1371/journal.pntd.0005660)
Supplement: S2 Table — (DOCX) [file pntd.0005660.s002.docx]

**S2 Table. Human seroprevalence of *Leishmania* and phleboviruses in the Mediterranean Region.**

| **Country** | ***Leishmania*** | | | | ***Phlebovirus*** | | | |
| --- | --- | --- | --- | --- | --- | --- | --- | --- |
|  | Seroprevalence/ Year^a^ | Study cohort | Test | Ref.^b^ | Seroprevalence/ Year^a^ | Study cohort | Test | Ref.^b^ |
| Spain | 44% (1996) | General population (Granada province) | LST | [1] | 15% anti-Granada virus IgG (2010) | General population (Granada province) | IFA | [2] |
|  | 52% (2002) | General population (L’Alacantì region) | LST | [3] | 3% anti-Granada virus NT-Ab (2013) | General population (Granada province) | NT | [4] |
|  | 5% (2004) | General population (Castilla-Leon region) | EIA | [5] | 12% anti-Naples virus IgG (1998) | Blood donors | IFA | [6] |
|  | 3% ^c^ (2008) | Blood donors (Balearic Islands) | WB | [7] | 2% anti-Sicilian virus IgG (1998) | Blood donors | IFA | [6] |
|  | 2% ^d^ (2011) | Blood donors (Murcia region) | EIA | [8] | 26% anti-TOSV IgG (1998) | Blood donors | IFA | [6] |
| France | 65% (1992) | General population (Marseille province) | WB | [9] | 1% anti-Sicilian virus IgG (2011) | General population (Marseille province) | IFA | [10] |
|  | 13% (1999) | Blood donors (Marseille province) | WB | [11] | 3% and 12% anti-TOSV IgM and IgG, respectively (2011) | Blood donors (southeastern France) | EIA | [12] |
|  | 28%^e^ (2011) | Hospitalized patients (Marseille province) | WB | [13] | 21.4%^e^ anti-TOSV IgG | Hospitalized patients (Marseille province) | IFA | [13] |
| Italy | 10% (1990) | General population (Sardinia) | LST | [14] | 9% anti-Sicilian virus IgG (2012) | General population (Sicily) | IFA | [15] |
|  | 3% and 4% (1991) | General population (Latium and Sicily, respectively) | IHA | [16] | 22% anti-TOSV IgG (2003) | General population (Tuscany) | EIA | [17] |
|  | 0.75% (2008) | Blood donors (Sicily) | IFA | [18] | 33% anti-TOSV IgG (2012) | General population (Sicily) | EIA | [15] |
|  | 7% ^f^ (2010) | General population (Piedmont) | WB | [19] |  |  |  |  |
| Greece | 15% (2003) | Blood donors (Crete) | WB+IFA | [20] | 4% anti-Corfou virus (1990) | General population | IFA | [21] |
|  | 3% (2008) | General population (northern Greece) | IFA | [22] | 17% anti-Naples virus NT-Ab (1990) | General population | NT | [21] |
|  |  |  |  |  | 2% anti-Sicilian virus NT-Ab (1990) | General population | NT | [21] |
|  |  |  |  |  | 39% and 52% anti-TOSV IgG (2010) | General population (Cephalonia and Corfù, respectively) | IFA+EIA | [23] |
|  |  |  |  |  | 22% anti-TOSV IgG (2013) | General population (Aegean Sea islands) | EIA | [24] |
|  |  |  |  |  | 0%-15% anti-TOSV IgG (2013) | General population (Greece mainland, different regions) | EIA | [25] |
| Balkans | 11% (2013) | General population (Croatia) | EIA | [26] | 0.01% anti-Naples virus NT-Ab (2011) | Blood donors (Kosovo) | NT | [27] |
|  |  |  |  |  | 0.01% anti-TOSV NT-Ab (2011) | Blood donors (Kosovo) | NT | [27] |
|  |  |  |  |  | 38% anti-TOSV IgG (2012) | General population (Croatia) | EIA | [28] |
| Cyprus |  |  |  |  | 57% anti-Naples virus NT-Ab (1991) | General population | NT | [29] |
|  |  |  |  |  | 32% anti-Sicilian virus NT-Ab (1991) | General population | NT | [29] |
|  |  |  |  |  | 20% anti-TOSV NT-Ab (1991) | General population | NT | [29] |
| Turkey | 6% (2012) | Blood donors | IFA | [30] | 0.7% anti-Adana virus NT-Ab (2015) | General population (Adana and Mersin province) | MN | [31] |
|  |  |  |  |  | 5% anti-Naples virus NT-Ab (2011) | Blood donors | NT | [32] |
|  |  |  |  |  | 15% anti-Sicilian virus NT-Ab (2011) | Blood donors | NT | [32] |
|  |  |  |  |  | 14% anti-TOSV NT-Ab (2011) | Blood donors | NT | [32] |
|  |  |  |  |  | 12% anti-Turkey virus NT-Ab (2011) | Blood donors | NT | [32] |
| Israel |  |  |  |  | 17% anti-Naples virus IgG (1999) | Healthy soldiers | EIA | [33] |
|  |  |  |  |  | 12% anti-Sicilian virus IgG (1999) | Healthy soldiers | EIA | [33] |
| Algeria |  |  |  |  | 21% anti-Naples virus IgG (2010) | General population (northern Algeria) | IFA+EIA | [34] |
|  |  |  |  |  | 5% anti-Sicilian virus IgG (2008) | General population | IFA | [35] |
|  |  |  |  |  | 50% anti-TOSV NT-Ab (2015) | Blood donors (Kabylia region) | MN | [36] |
| Tunisia |  |  |  |  | 1% anti-Medjerda Valley virus NT-Ab (2015) | General population (northern Tunisia) | MN | [37] |
|  |  |  |  |  | 9% anti-Punique virus NT-Ab (2013) | General population (governorate of Bizerte) | MN | [38] |
|  |  |  |  |  | 10% anti-TOSV IgG (2013) | Blood donors | EIA | [39] |
|  |  |  |  |  | 41% anti-TOSV NT-Ab (2013) | General population (governorate of Bizerte) | MN | [38] |
| Egypt |  |  |  |  | 2% anti-Naples virus IgG (1993) | General population (governorate of Sharqiya) | EIA | [40] |
|  |  |  |  |  | 4% anti-Sicilian virus IgG (1993) | General population (governorate of Sharqiya) | EIA | [40] |

^a^ Year of the article’s publication

^b^ Only data published after 1990 are reported.

^c^ 6% of tested donors were positive for *Leishmania* DNA in peripheral blood.

^d^ 8% of tested donors were positive for *Leishmania* DNA in blood [41].

^e^ This study reports the first scientific evidence for an epidemiological link between *L.infantum* and TOSV [13].

^f^ 4% of tested subjects were positive for *Leishmania* DNA in peripheral blood.

Ref: References; EIA: enzyme immunoassay; LST: Leishmania skin test; WB: Western Blotting; IHA: indirect haemoagglutination test; IFA: immunofluorescent assay; NT: neutralisation test; NT-Ab: neutralizing antibodies; MN: microneutralisation test

**References**

1. Acedo Sanchez C, Martin Sanchez J, Velez Bernal ID, Sanchis Marin MC, Louassini M, Maldonado JA, et al. Leishmaniasis Eco-epidemiology in the Alpujarra Region (Granada province, southern Spain). *International Journal for Parasitology*1996. p. 303-10.

2. Collao X, Palacios G, de Ory F, Sanbonmatsu S, Pérez-Ruiz M, Navarro JM, et al. Granada virus: a natural phlebovirus reassortant of the sandfly fever Naples serocomplex with low seroprevalence in humans. Am J Trop Med Hyg. 2010;83(4):760-5.

3. Moral L, Rubio EM, Moya M. A leishmanin skin test survey in the human population of L'Alacantì region (Spain): implications for the epidemiology of Leishmania infantum infection in southern Europe. Trans R Soc Trop Med Hyg. 2002;96:129-32.

4. Navarro-Marí JM, Gómez-Camarasa C, Pérez-Ruiz M, Sanbonmatsu-Gámez S, Pedrosa-Corral I, Jiménez-Valera M. Clinic-epidemiologic study of human infection by Granada virus, a new phlebovirus within the sandfly fever Naples serocomplex. Am J Trop Med Hyg. 2013;88(5):1003-6.

5. Garrote JI, Gutiérrez MP, Izquierdo RL, Dueñas MA, Zarzosa P, Cañavate C, et al. Seroepidemiologic study of Leishmania infantum infection in Castilla-Leon, Spain. Am J Trop Med Hyg. 2004;71(4):403-6.

6. Mendoza-Montero J, Gámez-Rueda MI, Navarro-Marí JM, de la Rosa-Fraile M, Oyonarte-Gómez S. Infections due to sandfly fever virus serotype Toscana in Spain. Clin Infect Dis. 1998;27(3):434-6.

7. Riera C, Fisa R, López-Chejade P, Serra T, Girona E, Jiménez M, et al. Asymptomatic infection by Leishmania infantum in blood donors from the Balearic Islands (Spain). Transfusion. 2008;48(7):1383-9.

8. Chitimia L, Muñoz-García CI, Sánchez-Velasco D, Lizana V, Del Río L, Murcia L, et al. Cryptic Leishmaniosis by Leishmania infantum, a feature of canines only? A study of natural infection in wild rabbits, humans and dogs in southeastern Spain. Vet Parasitol. 2011;181(1):12-6.

9. Mary C, Lamouroux D, Dunan S, Quilici M. Western blot analysis of antibodies to Leishmania infantum antigens: potential of the 14-kD and 16-kD antigens for diagnosis and epidemiologic purposes. Am J Trop Med Hyg. 1992;47(6):764-71.

10. Bichaud L, Piarroux RP, Izri A, Ninove L, Mary C, De Lamballerie X, et al. Low seroprevalence of sandfly fever Sicilian virus antibodies in humans, Marseille, France. Clin Microbiol Infect. 2011;17(8):1189-90.

11. le Fichoux Y, Quaranta JF, Aufeuvre JP, Lelievre A, Marty P, Suffia I, et al. Occurrence of Leishmania infantum parasitemia in asymptomatic blood donors living in an area of endemicity in southern France. J Clin Microbiol. 1999;37(6):1953-7.

12. Brisbarre N, Attoui H, Gallian P, Di Bonito P, Giorgi C, Cantaloube JF, et al. Seroprevalence of Toscana virus in blood donors, France, 2007. Emerg Infect Dis. 2011;17(5):941-3.

13. Bichaud L, Souris M, Mary C, Ninove L, Thirion L, Piarroux RP, et al. Epidemiologic relationship between Toscana virus infection and Leishmania infantum due to common exposure to Phlebotomus perniciosus sandfly vector. PLoS Negl Trop Dis. 2011;5(9):e1328.

14. Gramiccia M, Bettini S, Gradoni L, Ciarmoli P, Verrilli ML, Loddo S, et al. Leishmaniasis in Sardinia. 5. Leishmanin reaction in the human population of a focus of low endemicity of canine leishmaniasis. Trans R Soc Trop Med Hyg. 1990;84(3):371-4.

15. Calamusa G, Valenti RM, Vitale F, Mammina C, Romano N, Goedert JJ, et al. Seroprevalence of and risk factors for Toscana and Sicilian virus infection in a sample population of Sicily (Italy). J Infect. 2012;64(2):212-7.

16. Federico G, Damiano F, Caldarola G, Fantini C, Fiocchi V, Ortona L. A seroepidemiological survey on Leishmania infantum infection. Eur J Epidemiol. 1991;7(4):380-3.

17. Valassina M, Valentini M, Pugliese A, Valensin PE, Cusi MG. Serological survey of Toscana virus infections in a high-risk population in Italy. Clin Diagn Lab Immunol. 2003;10(3):483-4.

18. Scarlata F, Vitale F, Saporito L, Reale S, Vecchi VL, Giordano S, et al. Asymptomatic Leishmania infantum/chagasi infection in blood donors of western Sicily. Trans R Soc Trop Med Hyg. 2008;102(4):394-6.

19. Biglino A, Bolla C, Concialdi E, Trisciuoglio A, Romano A, Ferroglio E. Asymptomatic Leishmania infantum infection in an area of northwestern Italy (Piedmont region) where such infections are traditionally nonendemic. J Clin Microbiol. 2010;48(1):131-6.

20. Kyriakou DS, Alexandrakis MG, Passam FH, Kourelis TV, Foundouli P, Matalliotakis E, et al. Quick detection of Leishmania in peripheral blood by flow cytometry. Is prestorage leucodepletion necessary for leishmaniasis prevention in endemic areas? Transfus Med. 2003;13(2):59-62.

21. Antoniadis A, Alexiou-Daniel S, Malissiovas N, Doutsos I, Polyzoni T, LeDue JW, et al. Seroepidemiological survey for antibodies to arboviruses in Greece. Arch. Virol.1990. p. 277-85.

22. Diza E, Kansouzidou A, Gerou S, Vezyri E, Metallidis S, Antoniadis A. Leishmaniases in Northern Greece: seroprevalence of the infection and incidence of the disease during the period 2001-2006. Eur J Clin Microbiol Infect Dis. 2008;27(10):997-1003.

23. Papa A, Andriotis V, Tzilianos M. Prevalence of Toscana virus antibodies in residents of two Ionian islands, Greece. Travel Med Infect Dis. 2010;8(5):302-4.

24. Anagnostou V, Papa A. Seroprevalence of Toscana virus among residents of Aegean Sea islands, Greece. Travel Med Infect Dis. 2013;11(2):98-102.

25. Anagnostou V, Papa A. Prevalence of antibodies to phleboviruses within the sand fly fever Naples virus species in humans, northern Greece. Clin Microbiol Infect. 2013;19(6):566-70.

26. Šiško-Kraljević K, Jerončić A, Mohar B, Punda-Polić V. Asymptomatic Leishmania infantum infections in humans living in endemic and non-endemic areas of Croatia, 2007 to 2009. Euro Surveill. 2013;18(29):20533.

27. Venturi G, Marchi A, Fiorentini C, Ramadani N, Quaglio G, Kalaveshi A, et al. Prevalence of antibodies to phleboviruses and flaviviruses in Peja, Kosovo. Clin Microbiol Infect. 2011;17(8):1180-2.

28. Punda-Polić V, Jerončić A, Mohar B, Šiško Kraljević K. Prevalence of Toscana virus antibodies in residents of Croatia. Clin Microbiol Infect. 2012;18(6):E200-3.

29. Eitrem R, Stylianou M, Niklasson B. High prevalence rates of antibody to three sandfly fever viruses (Sicilian, Naples, and Toscana) among Cypriots. Epidemiol Infect. 1991;107(3):685-91.

30. Ates SC, Bagirova M, Allahverdiyev AM, Baydar SY, Koc RC, Elcicek S, et al. Detection of antileishmanial antibodies in blood sampled from blood bank donors in Istanbul. Future Microbiol. 2012;7(6):773-9.

31. Alkan C, Alwassouf S, Piorkowski G, Bichaud L, Tezcan S, Dincer E, et al. Isolation, genetic characterization, and seroprevalence of Adana virus, a novel phlebovirus belonging to the Salehabad virus complex, in Turkey. J Virol. 2015;89(8):4080-91.

32. Ergünay K, Saygan MB, Aydoğan S, Lo MM, Weidmann M, Dilcher M, et al. Sandfly fever virus activity in central/northern Anatolia, Turkey: first report of Toscana virus infections. Clin Microbiol Infect. 2011;17(4):575-81.

33. Cohen D, Zaide Y, Karasenty E, Schwarz M, LeDuc JW, Slepon R, et al. Prevalence of antibodies to West Nile fever, sandfly fever Sicilian, and sandfly fever Naples viruses in healthy adults in Israel. Public Health Rev. 1999;27(1-3):217-30.

34. Moureau G, Bichaud L, Salez N, Ninove L, Hamrioui B, Belazzoug S, et al. Molecular and serological evidence for the presence of novel phleboviruses in sandflies from northern Algeria. Open Virol J. 2010;4:15-21.

35. Izri A, Temmam S, Moureau G, Hamrioui B, de Lamballerie X, Charrel RN. Sandfly fever Sicilian virus, Algeria. Emerg Infect Dis. 2008;14(5):795-7.

36. Alkan C, Allal-Ikhlef AB, Alwassouf S, Baklouti A, Piorkowski G, de Lamballerie X, et al. Virus isolation, genetic characterization and seroprevalence of Toscana virus in Algeria. Clin Microbiol Infect. 2015;21(11):1040.e1-9.

37. Bichaud L, Dachraoui K, Alwassouf S, Alkan C, Mensi M, Piorkowski G, et al. Isolation, full genomic characterisation and neutralisation-based human seroprevalence of Medjerda Valley virus, a novel sandfly-borne phlebovirus belonging to the Salehabad virus complex in northern Tunisia. J Gen Virol. 2015.

38. Sakhria S, Bichaud L, Mensi M, Salez N, Dachraoui K, Thirion L, et al. Co-circulation of Toscana virus and Punique virus in northern Tunisia: a microneutralisation-based seroprevalence study. PLoS Negl Trop Dis. 2013;7(9):e2429.

39. Fezaa O, Bahri O, Alaya Bouafif NB, Triki H, Bouattour A. Seroprevalence of Toscana virus infection in Tunisia. Int J Infect Dis. 2013;17(12):e1172-5.

40. Corwin A, Habib M, Watts D, Darwish M, Olson J, Botros B, et al. Community-based prevalence profile of arboviral, rickettsial, and Hantaan-like viral antibody in the Nile River Delta of Egypt. Am J Trop Med Hyg. 1993;48(6):776-83.

41. Pérez-Cutillas P, Goyena E, Chitimia L, De la Rúa P, Bernal LJ, Fisa R, et al. Spatial distribution of human asymptomatic Leishmania infantum infection in southeast Spain: a study of environmental, demographic and social risk factors. Acta Trop. 2015;146:127-34.
